# Oxygen-Tolerant RAFT Polymerization Initiated by Living Bacteria

**DOI:** 10.1021/acsmacrolett.2c00372

**Published:** 2022-07-12

**Authors:** Mechelle R. Bennett, Cara Moloney, Francesco Catrambone, Federico Turco, Benjamin Myers, Katalin Kovacs, Philip J. Hill, Cameron Alexander, Frankie J. Rawson, Pratik Gurnani

**Affiliations:** ‡ Division of Regenerative Medicine and Cellular Therapies, School of Pharmacy, University of Nottingham, University Park Campus, Nottingham NG7 2RD, United Kingdom; § School of Medicine, BioDiscovery Institute, University of Nottingham, University Park Campus, Nottingham NG7 2RD, United Kingdom; ⊥ School of Life Sciences, BioDiscovery Institute, University of Nottingham, University Park Campus, Nottingham NG7 2RD, United Kingdom; # School of Pharmacy, BioDiscovery Institute, University of Nottingham, University Park Campus, Nottingham NG7 2RD, United Kingdom; ○ Division of Molecular Therapeutics, School of Pharmacy, University of Nottingham, University Park Campus, Nottingham NG7 2RD, United Kingdom; □ Division of Microbiology, Brewing and Biotechnology, School of Biosciences, University of Nottingham, Sutton Bonington Campus, Nottingham LE12 5RD, United Kingdom

## Abstract

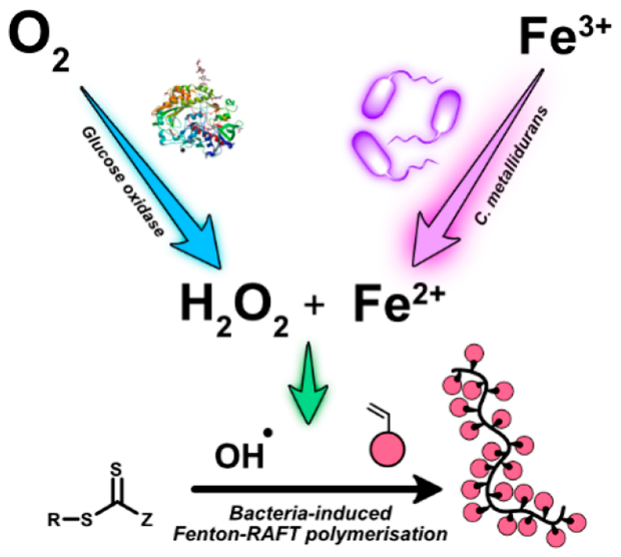

Living organisms can synthesize a wide range of macromolecules from a small set of natural building blocks, yet there is potential for even greater materials diversity by exploiting biochemical processes to convert unnatural feedstocks into new abiotic polymers. Ultimately, the synthesis of these polymers in situ might aid the coupling of organisms with synthetic matrices, and the generation of biohybrids or engineered living materials. The key step in biohybrid materials preparation is to harness the relevant biological pathways to produce synthetic polymers with predictable molar masses and defined architectures under ambient conditions. Accordingly, we report an aqueous, oxygen-tolerant RAFT polymerization platform based on a modified Fenton reaction, which is initiated by *Cupriavidus metallidurans* CH34, a bacterial species with iron-reducing capabilities. We show the synthesis of a range of water-soluble polymers under normoxic conditions, with control over the molar mass distribution, and also the production of block copolymer nanoparticles via polymerization-induced self-assembly. Finally, we highlight the benefits of using a bacterial initiation system by recycling the cells for multiple polymerizations. Overall, our method represents a highly versatile approach to producing well-defined polymeric materials within a hybrid natural-synthetic polymerization platform and in engineered living materials with properties beyond those of biotic macromolecules.

Nature exploits a vast array of biological pathways to produce biotic macromolecules (polysaccharides, proteins, DNA, RNA, etc.) derived from a small subset of monomers (e.g., sugars, amino acids, nucleobases, etc.). In contrast, the chemical industry has made available an enormous stock of monomers, particularly those with reactive double bonds, to provide routes to an almost limitless set of abiotic macromolecules. Polymers derived from vinylic or acrylic functionality have found use in biomedicine^1,2^ and as energy^3^ and information storage materials.^4,5^ Combining biosynthetic pathways with abiotic monomers could therefore generate an even greater diversity of materials and, if conducted in the presence of an organism with appropriate biochemical functionality, allow hybrid synthetic/natural interfaces and engineered living materials (ELMs) to be formed.

The cellular metabolism is underpinned by electron transport via redox pathways. We and others have shown that these pathways can be used in cell-activated polymerization.^6−11^ Prior reports have focused on the metal reducing activity of bacteria (e.g., *E. coli*, *C. metallidurans*, *S. oneidensis*) to mediate the active and dormant states of copper, iron and other metallic catalysts for atom transfer radical polymerizations (ATRP).^6−8,10^ However, ATRP suffers a disadvantage where the bacterial reduction kinetics directly control the balance of growing and dormant chains for desirable kinetics and molar mass distribution.^12^ In contrast, reversible addition–fragmentation chain transfer (RAFT) polymerization, which is a chain-transfer agent-mediated polymerization, requires instead a constant flux of external radicals. In many biological environments, a source of radicals is readily available, thus, RAFT might be inherently easier to control than cell-instructed ATRP, which is adversely affected by alternate indirect initiation pathways from bacterial cultures.^13^

While it has been shown that the generic reducing environment of bacteria can be used to produce organic radicals from the reduction of an aryl diazonium salt, which initiates the RAFT process,^11^ this has been achieved so far only under anoxic conditions, hindering the translation to biological applications. Conversely, many oxygen-tolerant RAFT polymerizations have been reported,^14^ either by polymerizing directly through oxygen^15−17^ or utilizing a scavenger such as an enzyme^18−20^ or oxygen trap,^21−25^ which has enabled ultralow reaction volumes,^17,19,22^ 3D/4D printing,^21,26^ and high-throughput platforms,^22^ but to the best of our knowledge have not been applied in a bacterially initiated RAFT polymerization.

Accordingly, in this study, we present a new oxygen-tolerant bacteria-initiated RAFT polymerization by utilizing an adapted Fenton polymerization.^27,28^ Our approach harnesses the substantially faster reaction rate (4–5 orders of magnitude) between hydrogen peroxide and Fe^2+^ than with Fe^3+^ to produce hydroxyl radicals to mediate the RAFT process. While a typical Fenton polymerization procedure directly implements Fe^2+^ to avoid this, we postulated that we could use the Fe^3+^ reducing capabilities of *C. metallidurans* CH34 metabolism, which instructs the in situ formation of Fe^2+^ and accelerates the formation of hydroxyl radicals to initiate the RAFT process. To achieve oxygen tolerance, we were inspired by previous studies that utilized glucose oxidase (GOx) to deoxygenate transiently the reaction media from a glucose feedstock.^18,19^ This approach provided a dual benefit, as a key byproduct from GOx deoxygenation is hydrogen peroxide which could be fed into our bacterially instructed Fenton reaction (Scheme 1).^29^ Using this approach, we report the optimization and mechanistic evaluation of our bacterially mediated Fenton polymerization. We highlight this through the synthesis of a range of well-defined RAFT polymers and polymer nanoparticles in open-to-air vessels under aqueous conditions.

**Scheme 1:**
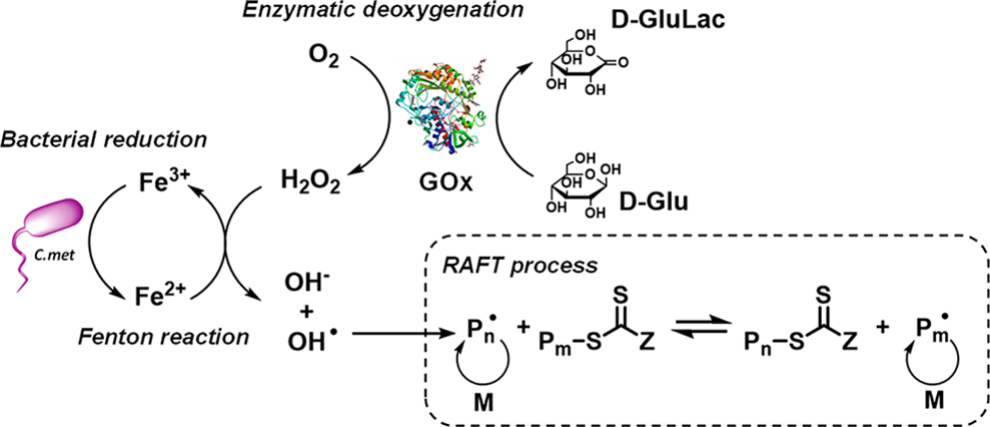
Fenton GOx RAFT Process Initiated by Bacteria d-Glucose (DG) is converted to d-glucanolactate (DGA) by glucose oxidase (GOx), which consumes O_2_ in the process to form H_2_O_2_. Without the presence of reducing agents, polymerization should not take place. GOx protein image from PDB ID: 3QVP.

Before conducting our bacteria mediated Fenton RAFT polymerizations, we initially evaluated the viability of *C. metallidurans* CH34 cells in the presence of a range of water-soluble monomers to ensure any observable polymerization was not caused by cell lysis (Figure S1 and Table S1). Both *N*,*N*-dimethylacrylamide (DMA) and *N*-hydroxyethylacrylamide (HEA) exhibited an MIC_50_ above 100 mM. However, *N*-acryloyl morpholine (NAM) displayed some toxicity toward the bacterial cultures (MIC_50_ = 42.5 mM). As a result of this, a concentration of 25 mM NAM was employed as this ensured *c*. 70% bacterial viability, a similar viability was observed at a monomer concentration of 100 mM for DMA and HEA.

To test our bacteria-instructed Fenton-RAFT hypothesis, we incubated a mixture of DMA monomer, carboxyethyl propanoic acid trithiocarbonate (CEPTC) water-soluble RAFT agent, FeCl_3_ as the Fe^3+^ source, glucose oxidase and glucose with a *C. metallidurans* culture (1.7 × 10^10^ colony forming units (CFU) mL^–1^) in phosphate-buffered saline (PBS) ([DMA]/[CTA]/[FeCl_3_]/[GOx]/[glucose] = 400:1:5.3:0.002:0.8) and heated the suspension to 30 °C in an open to air vessel for 24 h. Aside from its iron-reducing properties, *C. metallidurans* lacks the glucose transporter, thus, we deemed it unlikely that the bacterial cells were reducing the glucose concentration through metabolization.^30^ Conducting the polymerizations in PBS instead of growth medium also mitigated the risk of incorporating additional reducing agents, which may contribute to redox-based radical initiation pathways. After removal of the bacteria and iron oxide precipitate, ^1^H NMR spectroscopy confirmed the presence of polymer, with monomer conversion reaching 53% (Figure 1a).

**Figure 1:**
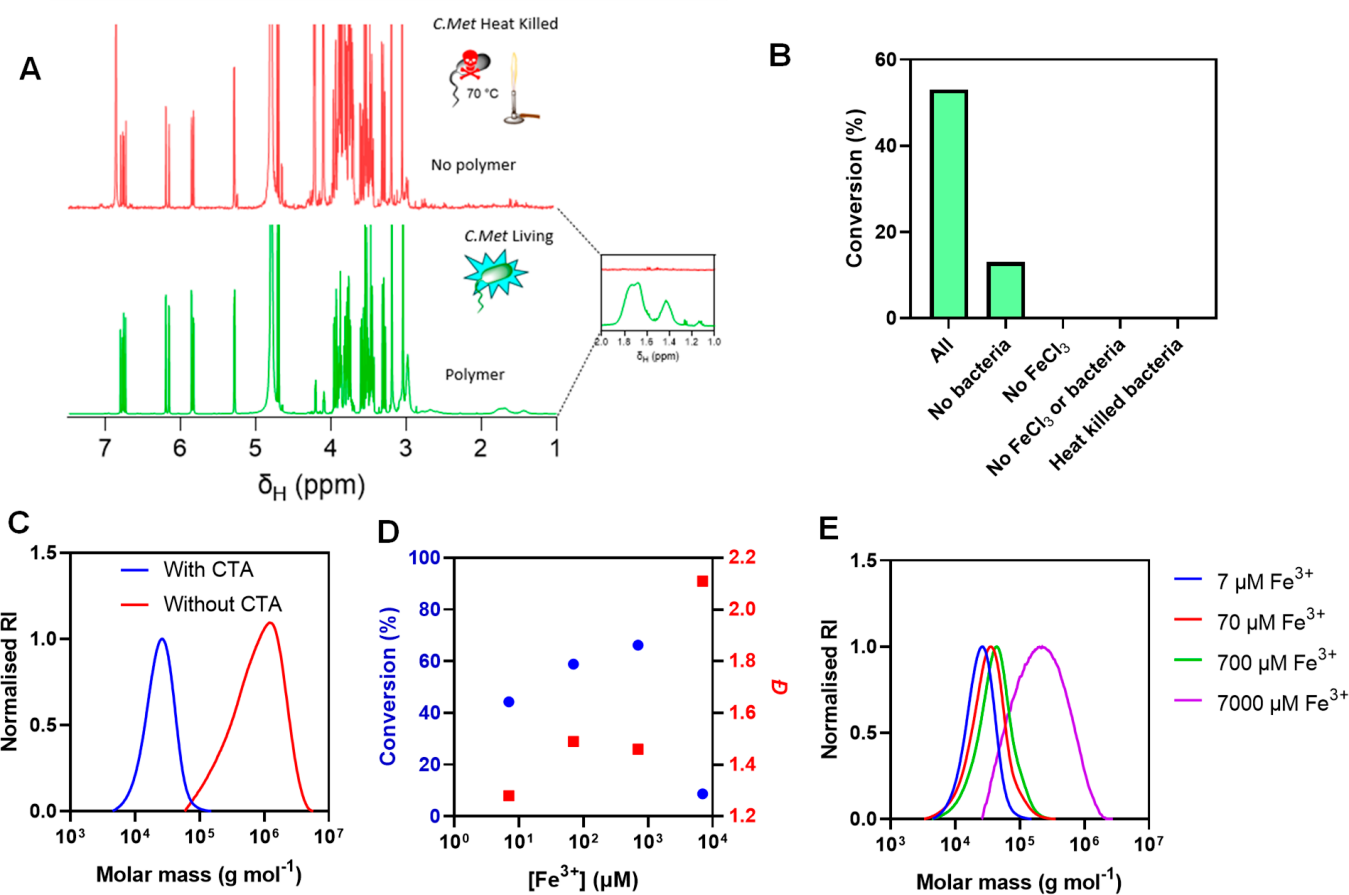
(a) ^1^H NMR stacked spectra of bacterial initiated polymerizations of DMA in air at 30 °C with either living *C. metallidurans* (bottom, green) and heat killed *C. metallidurans* (top, red). (b) Conversion as calculated by ^1^H NMR (400 MHz, D_2_O) of the final time point (20 h) in bacterial-initiated polymerizations showing the need for live bacteria and a Fe^3+^ source for high conversion polymerization to occur. (c) SEC (DMF) overlay of polymers produced with and without the addition of CTA. (d) Effect of concentration of Fe^3+^ on conversion from ^1^H NMR (400 MHz, D_2_O) and *Đ* from SEC (DMF). (e) Corresponding SEC (DMF, RI detector).

Size exclusion chromatography (SEC) analysis indicated a monomodal molecular weight distribution with low dispersity (*Đ* = 1.12) and low molar mass (*M*_n,SEC_ = 19900 g mol^–1^), as is expected for RAFT polymerization. Crucially, control experiments omitting FeCl_3_ or with *C. metallidurans* cultures, which were heat killed (3.6 × 10^2^ CFU mL^–1^), displayed no monomer conversion, indicating the importance of metabolically active cells for successful polymerization (Table S3). Noticeably, reaction mixtures containing FeCl_3_, but in the absence of bacteria, yielded a small level of polymerization (10% monomer conversion), which we suspect is due to the slower Fe^3+^-mediated Fenton reaction, producing a low concentration of hydroxyl radicals, which still contribute to conversion (Figures 1b and S2). Polymerizations in the absence of CTA yielded substantially higher molar masses (*M*_n,SEC_ = 451000 g mol^–1^) and high dispersity (*Đ* = 2.11) following a conventional free radical mechanism (Figure 1c).

When hydroxyl radicals are generated from the bacterially produced Fe^2+^, Fe^3+^ is regenerated during the Fenton reaction. We therefore postulated that the bacteria could recycle the available Fe^3+^ for further Fenton polymerizations at a reduced FeCl_3_ concentration. Accordingly, the pDMA produced in polymerizations conducted at 7 μM maintained narrow dispersities (*Đ* ∼ 1.28, Figure 1d) and still achieved moderate monomer conversions (44%). There was an increasing trend correlating FeCl_3_ concentration with monomer conversion between 7 and 700 μM, reaching a maximum of 66.2%, also resulting in an increase in *Đ* from 1.28 to 1.49. All polymers had unimodal molar mass distributions with similar *M*_n,SEC_ to their *M*_n,th_ values (Figure 1e). Strikingly, at 7 mM we observed a substantial reduction in monomer conversion to 9%, much broader molar mass distributions (*Đ* = 2.11) and *M*_n,SEC_ 50-fold higher than the *M*_n,th_ which is more consistent with free radical polymerization, likely caused by excess oxidation of the free RAFT agent and possible toxicity toward *C. metallidurans*.^31^ For this reason, we adopted Fe concentrations of 7 μM for the remaining experiments.

One of the key hallmarks of RAFT polymerizations is the ability to control the chain length and molar mass of the resulting macromolecules, hence, we examined if this feature was translatable to our bacterially assisted polymerizations. We conducted DMA polymerizations targeting three chain lengths, DP100, DP400, and DP800, by modifying the CTA concentration but maintaining the same conditions for all other reactants (Table S3). As expected for reversible deactivation radical polymerizations (RDRP), we observed a larger *M*_n,SEC_ for higher target DP (6300, 20700, and 55800 g mol^–1^ for DP100, DP400, and DP800, respectively; Figure S3A,B). Notably, we observed broader molar mass distributions (*Đ* = 1.7) for the DP800 DMA polymerization, suggesting some loss of control for larger chain lengths. We anticipate this may be due to a significantly lower apparent [CTA]/[I] at lower CTA concentrations, increasing the likelihood of termination of growing chains and thus RAFT agent loss.

Bacteria-assisted Fenton RAFT polymerizations with HEA and NAM (conducted at 100 and 25 mM monomer solutions, respectively) displayed similar monomer conversions to DMA (37 and 40% respectively), albeit with higher dispersities (*Đ* ∼ 1.6 for both polymerizations, compared to 1.28 for DMA; Figure 2a and Table S3). Although HEA polymers displayed moderately similar experimental and theoretical molar masses, the NAM analogues were 10-fold higher in molar mass than expected, attributed either due to the difference in monomer concentration or the poorer cell tolerability described above. To probe this, we performed a copolymerization of 20% NAM and 80% DMA (total monomer concentration = 62.5 mM), which produced a copolymer with similar experimental and theoretical molar masses and low dispersity (*Đ* = 1.21), suggesting this was due to the overall monomer concentration and not NAM toxicity.

**Figure 2:**
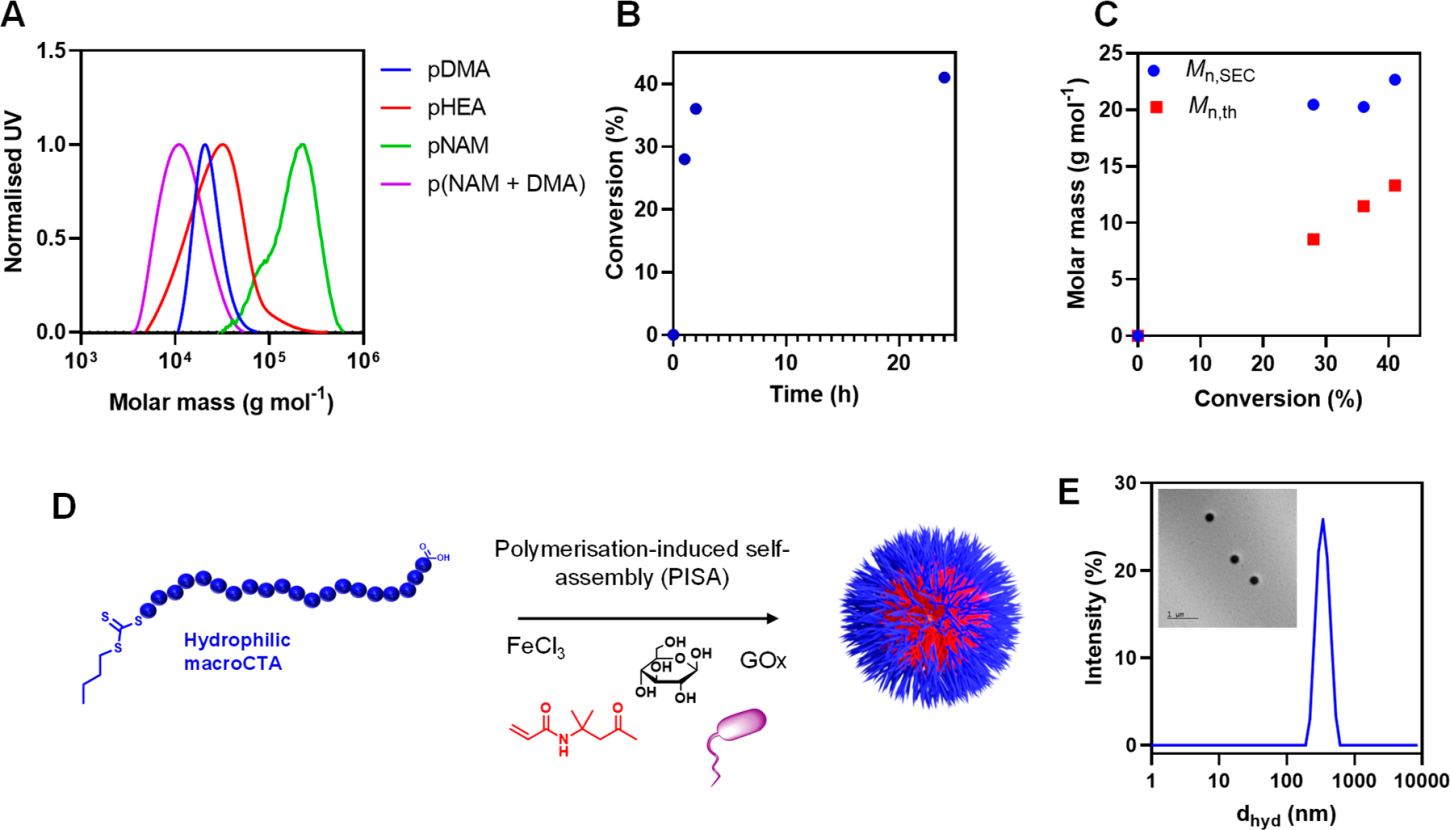
(a) SEC (aqueous, UV detector) of polymers prepared with varying monomers. (b) ^1^H NMR (400 MHz, D_2_O) kinetic plot showing the effect of polymerization time on the conversion. (c) Comparison of *M*_n,SEC_ (aqueous, UV signal) and *M*_n,th_ as a function of conversion calculated by ^1^H NMR (400 MHz, D_2_O). (d) Schematic representation of bacteria-initiated polymerization-induced self-assembly to form spherical polymer nanoparticles. (e) Recorded DLS data for nanoparticles formed by PISA; Inset: representative TEM image.

Following this, chain extension with 400 units of DMA from a pDMA_75_ macromolecular chain transfer agent (mCTA), previously synthesized through conventional RAFT polymerization, was attempted. While 49% monomer conversion and a visible increase in *M*_n,SEC_ was observed (from 8000 to 20200 g mol^–1^), the chromatogram revealed a bimodal distribution suggesting some extension but a poor blocking efficiency from the mCTA (Figure S4). As the higher molar mass peak retains some absorption at 309 nm, we were confident this population possessed the trithiocarbonate group at the chain end, however, we anticipate the poor blocking efficiency may be due to a chain length effect causing retardation of the chain transfer process, partial oxidation of the trithiocarbonate chain end or some adhesion of bacterially synthesized polymers to the cell surface as we have previously identified.^10^ It is also possible that a high level of termination occurs, leading to RAFT agent loss; however, in this case it is unclear why a high proportion of the non-chain-extended mCTA retains absorpotion at 309 nm, indicative of trithiocarbonate retention.

We then investigated the polymerization kinetics of our bacteria-initiated RAFT polymerization by sampling a DMA polymerization at 1, 2, and 24 h, monitoring monomer conversion and *M*_n,SEC_. Notably, we observed the polymerization did not proceed above 41% monomer conversion under these conditions (Figure 2b). This conversion is in line with other bacterial radical polymerization systems,^7,8,11^ and we anticipate it is due to the low initial monomer conversion, which quickly depletes, retarding the ensuing polymerization reaction, compounded by the consumption of the glucose feedstock by GOx. Although a uniform molar mass distribution (*Đ* < 1.40) and retention of the trithiocarbonate was observed across all time points, indicating contribution by the chain transfer agent (Figure S5b), only partial linear evolution between *M*_n,SEC_ and monomer conversion for RAFT polymerizations was observed, suggesting some RAFT characteristics.(Table S3 and Figure 2c). This is supported by the first-order kinetic plot (Figure S5), which indicates a fast linear reaction between 0 and 2 h, which then reached a plateau after 35% monomer conversion (Figure S5a). Although the relatively low monomer conversion of this polymerization is a potential limitation, the necessity for active metabolism and living cells to initiate polymerization, a notable difference compared to previous strategies,^11^ means conversion is correlated to the tolerability of the chosen monomers. A limitation in this experiment was the relatively small number of time point samples we were able to retrieve from the polymerization mixture due to the fast rate of reaction in the initial phases and the requirement to remove bacterial cells to inhibit the polymerization. Attempts to use traditional radical quenchers (e.g., hydroquinone) were unsuccessful, likely due to their activity being reliant on dissolved oxygen, which is not present in our system due to the enzymatic deoxygenation mechanism.

One of the major advantages of RAFT polymerizations is the ability to prepare block copolymer nanoparticles with relative ease,^32^ which have enormous potential in drug delivery^33^ and other applications.^34^ An extremely versatile route that has been explored for the past decade is the polymerization-induced self-assembly (PISA), enabling the preparation of well-defined nanoparticles in situ during the polymerization which can be conducted under completely aqueous conditions (Figure 2d).^35,36^ Given the success of this approach and our encouraging results with bacteria-initiated solution polymerizations, we explored if we could utilize the methodology presented here to produce block copolymer nanoparticles via PISA. The pDMA_75_ mCTA was extended with a target 200 units of diacetone acrylamide, a monomer known to undergo PISA,^37−39^ reaching quantitative monomer conversion as is expected in PISA due to the high local monomer concentration within the growing particles. Particle size analysis via both DLS and TEM indicates successful nanoparticle preparation with corroborative sizes between the two techniques (Figure 2e, *Z*-average diameter = 345 nm). However, due to the low concentrations used in our PISA reaction no molar mass information could be obtained from dried particles. The ability to produce nanoparticles using this system could in the future offer the potential for biomimetic extracellular vesicles, which are achievable through PISA,^40^ which could for instance transport innate quorum-sensing molecules.^41^

A key benefit of utilizing living systems to initiate chemical reactions or indeed polymerizations is their ability to be reused or expanded through culture to remove feedstock requirements, important for the sustainability of these processes. Hence, we subsequently investigated if the initial *C. metallidurans* culture could be recycled for several polymerization reactions by pelleting the cells through centrifugation and resuspension with a new polymerization mixture (Figure 3a). It was found that the initial bacterial culture could be reused at least three times using without supplementing with growth media or nutrients. Interestingly the monomer conversion and *M*_n,SEC_ was variable between each cycle at 40, 80, and 50% for the three consecutive polymerizations and 18800, 32500, and 26500 g mol^–1^, respectively, each with low *Đ* (*Đ* ∼ 1.3) in all cases. While further investigation is required to understand fully these differences, we anticipate that some bacterial proliferation or changes in bacterial metabolism may affect final conversion. (Figure 3b,c).

**Figure 3:**
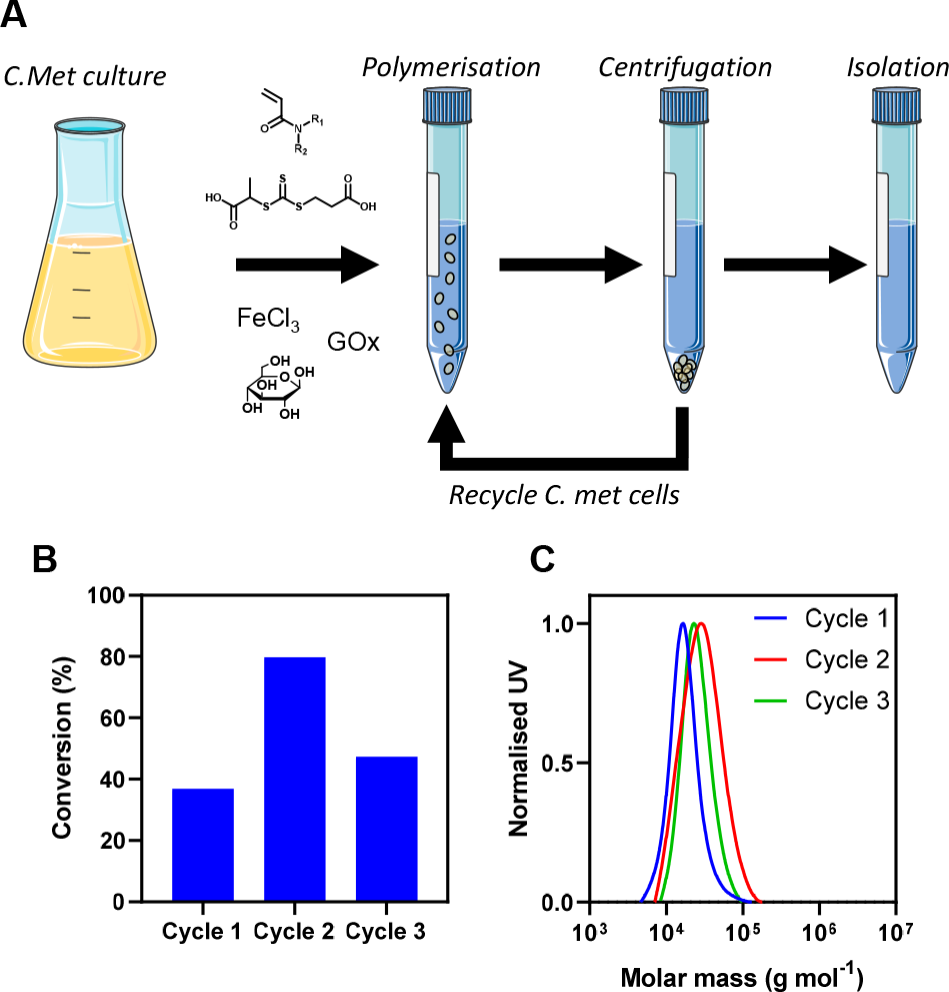
(a) Schematic representation of bacteria recycling. (b) Monomer conversion of polymerizations with bacteria recycling as determined by ^1^H NMR (400 MHz, D_2_O). (c) Corresponding SEC chromatograms (aqueous, UV detector). The figure was partly generated using Servier Medical Art, provided by Servier, licensed under a Creative Commons Attribution 3.0 unported license.

A similar phenomenon was reported by Keitz and co-workers for the bacteria mediated Cu(I)-catalyzed azide–alkyne cycloaddition, where subsequent cycles yielded different reaction conversions to the first cycle, which they suggested was due to bacterial growth or a change in growth phase between cycles 1 and 2.^42^

In conclusion, we have developed an oxygen-tolerant bacterially initiated polymerization method that can be used to produce macromolecules with a defined length via RAFT polymerization. To achieve this, we utilized the reducing capabilities of *C. metallidurans* to produce Fe^2+^ in situ and a simultaneous glucose oxide catalysis pathway to generate hydrogen peroxide from a glucose feedstock, which then reacts to produce hydroxyl radicals and initiate polymerization. We found that high monomer conversion could only be achieved with actively metabolizing bacteria and in the presence of Fe^3+^, supporting our proposed mechanism. Synthesized polymers exhibited the characteristics of conventional RAFT polymerizations such as narrow molecular weight distributions, retention of end-group fidelity and similar average molar masses, albeit with some limits in terms of blocking efficiencies. We exemplified this polymerization technique by utilizing monomers known to undergo polymerization-induced self-assembly to produce bacterially synthesized polymer nanoparticles. Finally, we showcased the ability for the bacteria to be a reusable component for radical generation and thus polymerization. This microbial redox pathway to produce well-defined polymers could open the potential for hybrid natural and non-natural material platforms and thus new engineered living materials.

## Supporting Information Available

Relevant methodology and characterization information for this work (PDF)

## References

[ref1] Hook A. L.; Chang C.-Y.; Yang J.; Luckett J.; Cockayne A.; Atkinson S.; Mei Y.; Bayston R.; Irvine D. J.; Langer R.; Anderson D. G.; Williams P.; Davies M. C.; Alexander M. R. Combinatorial discovery of polymers resistant to bacterial attachment. Nat. Biotechnol. 2012, 30 (9), 868–875. 10.1038/nbt.2316. 22885723 PMC3796337

[ref2] Kuroki A.; Sangwan P.; Qu Y.; Peltier R.; Sanchez-Cano C.; Moat J.; Dowson C. G.; Williams E. G. L.; Locock K. E. S.; Hartlieb M.; Perrier S. Sequence Control as a Powerful Tool for Improving the Selectivity of Antimicrobial Polymers. ACS Appl. Mater. Interfaces 2017, 9 (46), 40117–40126. 10.1021/acsami.7b14996. 29068226

[ref3] Hansen K.-A.; Blinco J. P. Nitroxide radical polymers – a versatile material class for high-tech applications. Polym. Chem. 2018, 9 (13), 1479–1516. 10.1039/C7PY02001E.

[ref4] Szweda R.; Tschopp M.; Felix O.; Decher G.; Lutz J.-F. Sequences of Sequences: Spatial Organization of Coded Matter through Layer-by-Layer Assembly of Digital Polymers. Angew. Chem., Int. Ed. 2018, 57 (48), 15817–15821. 10.1002/anie.201810559. 30290053

[ref5] Lee J. M.; Koo M. B.; Lee S. W.; Lee H.; Kwon J.; Shim Y. H.; Kim S. Y.; Kim K. T. High-density information storage in an absolutely defined aperiodic sequence of monodisperse copolyester. Nat. Commun. 2020, 11 (1), 56 10.1038/s41467-019-13952-2. 31911612 PMC6946701

[ref6] Bennett M. R.; Gurnani P.; Hill P. J.; Alexander C.; Rawson F. J. Iron-Catalysed Radical Polymerisation by Living Bacteria. Angew. Chem., Int. Ed. 2020, 59 (12), 4750–4755. 10.1002/anie.201915084. 31894618

[ref7] Fan G.; Dundas C. M.; Graham A. J.; Lynd N. A.; Keitz B. K. Shewanella oneidensis as a living electrode for controlled radical polymerization. Proc. Natl. Acad. Sci. U. S. A. 2018, 115 (18), 4559–4564. 10.1073/pnas.1800869115. 29666254 PMC5939106

[ref8] Fan G.; Graham A. J.; Kolli J.; Lynd N. A.; Keitz B. K. Aerobic radical polymerization mediated by microbial metabolism. Nat. Chem. 2020, 12 (7), 638–646. 10.1038/s41557-020-0460-1. 32424254 PMC7321916

[ref9] Luo Y.; Gu Y.; Feng R.; Brash J.; Eissa A. M.; Haddleton D. M.; Chen G.; Chen H. Synthesis of glycopolymers with specificity for bacterial strains via bacteria-guided polymerization. Chemical Science 2019, 10 (20), 5251–5257. 10.1039/C8SC05561K. 31191880 PMC6540911

[ref10] Magennis E. P.; Fernandez-Trillo F.; Sui C.; Spain S. G.; Bradshaw D. J.; Churchley D.; Mantovani G.; Winzer K.; Alexander C. Bacteria-instructed synthesis of polymers for self-selective microbial binding and labelling. Nat. Mater. 2014, 13 (7), 748–755. 10.1038/nmat3949. 24813421 PMC4286827

[ref11] Nothling M. D.; Cao H.; McKenzie T. G.; Hocking D. M.; Strugnell R. A.; Qiao G. G. Bacterial Redox Potential Powers Controlled Radical Polymerization. J. Am. Chem. Soc. 2021, 143 (1), 286–293. 10.1021/jacs.0c10673. 33373526

[ref12] Ouchi M.; Sawamoto M. 50th Anniversary Perspective: Metal-Catalyzed Living Radical Polymerization: Discovery and Perspective. Macromolecules 2017, 50 (7), 2603–2614. 10.1021/acs.macromol.6b02711.

[ref13] Perrier S. 50th Anniversary Perspective: RAFT Polymerization—A User Guide. Macromolecules 2017, 50 (19), 7433–7447. 10.1021/acs.macromol.7b00767.

[ref14] Yeow J.; Chapman R.; Gormley A. J.; Boyer C. Up in the air: oxygen tolerance in controlled/living radical polymerisation. Chem. Soc. Rev. 2018, 47 (12), 4357–4387. 10.1039/C7CS00587C. 29718038 PMC9857479

[ref15] Gody G.; Barbey R.; Danial M.; Perrier S. Ultrafast RAFT polymerization: multiblock copolymers within minutes. Polym. Chem. 2015, 6 (9), 1502–1511. 10.1039/C4PY01251H.

[ref16] Gurnani P.; Floyd T.; Tanaka J.; Stubbs C.; Lester D.; Sanchez-Cano C.; Perrier S. PCR-RAFT: rapid high throughput oxygen tolerant RAFT polymer synthesis in a biology laboratory. Polym. Chem. 2020, 11 (6), 1230–1236. 10.1039/C9PY01521C.

[ref17] Tanaka J.; Gurnani P.; Cook A. B.; Häkkinen S.; Zhang J.; Yang J.; Kerr A.; Haddleton D. M.; Perrier S.; Wilson P. Microscale synthesis of multiblock copolymers using ultrafast RAFT polymerisation. Polym. Chem. 2019, 10 (10), 1186–1191. 10.1039/C8PY01437J.

[ref18] Chapman R.; Gormley A. J.; Herpoldt K.-L.; Stevens M. M. Highly Controlled Open Vessel RAFT Polymerizations by Enzyme Degassing. Macromolecules 2014, 47 (24), 8541–8547. 10.1021/ma5021209.

[ref19] Chapman R.; Gormley A. J.; Stenzel M. H.; Stevens M. M. Combinatorial Low-Volume Synthesis of Well-Defined Polymers by Enzyme Degassing. Angew. Chem., Int. Ed. 2016, 55 (14), 4500–4503. 10.1002/anie.201600112. 26939064

[ref20] Nothling M. D.; Fu Q.; Reyhani A.; Allison-Logan S.; Jung K.; Zhu J.; Kamigaito M.; Boyer C.; Qiao G. G. Progress and Perspectives Beyond Traditional RAFT Polymerization. Advanced Science 2020, 7 (20), 2001656 10.1002/advs.202001656. 33101866 PMC7578854

[ref21] Bagheri A.; Bainbridge C. W. A.; Engel K. E.; Qiao G. G.; Xu J.; Boyer C.; Jin J. Oxygen Tolerant PET-RAFT Facilitated 3D Printing of Polymeric Materials under Visible LEDs. ACS Applied Polymer Materials 2020, 2 (2), 782–790. 10.1021/acsapm.9b01076.

[ref22] Gormley A. J.; Yeow J.; Ng G.; Conway Ó.; Boyer C.; Chapman R. An Oxygen-Tolerant PET-RAFT Polymerization for Screening Structure–Activity Relationships. Angew. Chem., Int. Ed. 2018, 57 (6), 1557–1562. 10.1002/anie.201711044. PMC9641662 29316089

[ref23] Ng G.; Yeow J.; Xu J.; Boyer C. Application of oxygen tolerant PET-RAFT to polymerization-induced self-assembly. Polym. Chem. 2017, 8 (18), 2841–2851. 10.1039/C7PY00442G.

[ref24] Wu C.; Jung K.; Ma Y.; Liu W.; Boyer C. Unravelling an oxygen-mediated reductive quenching pathway for photopolymerisation under long wavelengths. Nat. Commun. 2021, 12 (1), 478 10.1038/s41467-020-20640-z. 33473121 PMC7817663

[ref25] Xu J.; Jung K.; Boyer C. Oxygen Tolerance Study of Photoinduced Electron Transfer–Reversible Addition–Fragmentation Chain Transfer (PET-RAFT) Polymerization Mediated by Ru(bpy)3Cl2. Macromolecules 2014, 47 (13), 4217–4229. 10.1021/ma500883y.

[ref26] Zhang Z.; Corrigan N.; Bagheri A.; Jin J.; Boyer C. A Versatile 3D and 4D Printing System through Photocontrolled RAFT Polymerization. Angew. Chem., Int. Ed. 2019, 58 (50), 17954–17963. 10.1002/anie.201912608. 31642580

[ref27] Reyhani A.; McKenzie T. G.; Fu Q.; Qiao G. G. Fenton-Chemistry-Mediated Radical Polymerization. Macromol. Rapid Commun. 2019, 40 (18), 1900220 10.1002/marc.201900220. 31259456

[ref28] Reyhani A.; McKenzie T. G.; Ranji-Burachaloo H.; Fu Q.; Qiao G. G. Fenton-RAFT Polymerization: An “On-Demand” Chain-Growth Method. Chem. Eur. J. 2017, 23 (30), 7221–7226. 10.1002/chem.201701410. 28382790

[ref29] Reyhani A.; Nothling M. D.; Ranji-Burachaloo H.; McKenzie T. G.; Fu Q.; Tan S.; Bryant G.; Qiao G. G. Blood-Catalyzed RAFT Polymerization. Angew. Chem., Int. Ed. 2018, 57 (32), 10288–10292. 10.1002/anie.201802544. 29920886

[ref30] Van Houdt R.; Provoost A.; Van Assche A.; Leys N.; Lievens B.; Mijnendonckx K.; Monsieurs P. Cupriavidus metallidurans Strains with Different Mobilomes and from Distinct Environments Have Comparable Phenomes. Genes 2018, 9 (10), 507 10.3390/genes9100507. PMC6210171 30340417

[ref31] Jesson C. P.; Pearce C. M.; Simon H.; Werner A.; Cunningham V. J.; Lovett J. R.; Smallridge M. J.; Warren N. J.; Armes S. P. H2O2 Enables Convenient Removal of RAFT End-Groups from Block Copolymer Nano-Objects Prepared via Polymerization-Induced Self-Assembly in Water. Macromolecules 2017, 50 (1), 182–191. 10.1021/acs.macromol.6b01963. 31007283 PMC6471490

[ref32] Keddie D. J. A guide to the synthesis of block copolymers using reversible-addition fragmentation chain transfer (RAFT) polymerization. Chem. Soc. Rev. 2014, 43 (2), 496–505. 10.1039/C3CS60290G. 24129793

[ref33] Cabral H.; Miyata K.; Osada K.; Kataoka K. Block Copolymer Micelles in Nanomedicine Applications. Chem. Rev. 2018, 118 (14), 6844–6892. 10.1021/acs.chemrev.8b00199. 29957926

[ref34] Feng H.; Lu X.; Wang W.; Kang N.-G.; Mays J. W. Block Copolymers: Synthesis, Self-Assembly, and Applications. Polymers 2017, 9 (10), 494 10.3390/polym9100494. PMC6418972 30965798

[ref35] Penfold N. J. W.; Yeow J.; Boyer C.; Armes S. P. Emerging Trends in Polymerization-Induced Self-Assembly. ACS Macro Lett. 2019, 8 (8), 1029–1054. 10.1021/acsmacrolett.9b00464. 35619484

[ref36] Warren N. J.; Armes S. P. Polymerization-Induced Self-Assembly of Block Copolymer Nano-objects via RAFT Aqueous Dispersion Polymerization. J. Am. Chem. Soc. 2014, 136 (29), 10174–10185. 10.1021/ja502843f. 24968281 PMC4111214

[ref37] Byard S. J.; Williams M.; McKenzie B. E.; Blanazs A.; Armes S. P. Preparation and Cross-Linking of All-Acrylamide Diblock Copolymer Nano-Objects via Polymerization-Induced Self-Assembly in Aqueous Solution. Macromolecules 2017, 50 (4), 1482–1493. 10.1021/acs.macromol.6b02643. 28260814 PMC5333187

[ref38] He J.; Xu Q.; Tan J.; Zhang L. Ketone-Functionalized Polymer Nano-Objects Prepared via Photoinitiated Polymerization-Induced Self-Assembly (Photo-PISA) Using a Poly(diacetone acrylamide)-Based Macro-RAFT Agent. Macromol. Rapid Commun. 2019, 40 (2), 1800296 10.1002/marc.201800296. 29947031

[ref39] Rho J. Y.; Scheutz G. M.; Häkkinen S.; Garrison J. B.; Song Q.; Yang J.; Richardson R.; Perrier S.; Sumerlin B. S. In situ monitoring of PISA morphologies. Polym. Chem. 2021, 12 (27), 3947–3952. 10.1039/D1PY00239B.

[ref40] Blackman L. D.; Varlas S.; Arno M. C.; Fayter A.; Gibson M. I.; O’Reilly R. K. Permeable Protein-Loaded Polymersome Cascade Nanoreactors by Polymerization-Induced Self-Assembly. ACS Macro Lett. 2017, 6 (11), 1263–1267. 10.1021/acsmacrolett.7b00725. 29226025 PMC5718297

[ref41] Toyofuku M.; Morinaga K.; Hashimoto Y.; Uhl J.; Shimamura H.; Inaba H.; Schmitt-Kopplin P.; Eberl L.; Nomura N. Membrane vesicle-mediated bacterial communication. ISME Journal 2017, 11 (6), 1504–1509. 10.1038/ismej.2017.13. 28282039 PMC5437348

[ref42] Partipilo G.; Graham A. J.; Belardi B.; Keitz B. K. Extracellular Electron Transfer Enables Cellular Control of Cu(I)-Catalyzed Alkyne–Azide Cycloaddition. ACS Central Science 2022, 8, 246–257. 10.1021/acscentsci.1c01208. 35233456 PMC8875427

