# Supplementary material for: Oxygen-Tolerant RAFT Polymerization Initiated by Living Bacteria
Source: ACS Macro Lett. 2022 Jul 12;11(8):954–60. doi: 10.1021/acsmacrolett.2c00372 (PMC9387098; doi:10.1021/acsmacrolett.2c00372)
Supplement: Supplementary file 1 — mz2c00372_si_001.pdf [file mz2c00372_si_001.pdf]

Supplementary information for:

Oxygen tolerant RAFT polymerisation initiated by living bacteria

Mechelle R. Bennett‡, Cara Moloney‡, Francesco Catrambone§, Federico Turco§, Benjamin Myers, Katalin Kovács, Phillip J. Hill, Cameron Alexander, Frankie J. Rawson*, Pratik Gurnani*

Corresponding Authors

P. Gurnani - Division of Molecular Therapeutics and Formulation, School of Pharmacy, University of Nottingham, University Park Campus, Nottingham NG7 2RD (UK). Email: Pratik.gurnani@nottingham.ac.uk

F. J. Rawson - Division of Regenerative Medicine and Cellular Therapies, School of Pharmacy, University of Nottingham, University Park Campus, Nottingham NG7 2RD (UK). Email: Frankie.Rawson@nottingham.ac.uk

Authors

M. R. Bennett, - Division of Regenerative Medicine and Cellular Therapies, School of Pharmacy, University of Nottingham, University Park Campus, Nottingham NG7 2RD (UK)

C. Moloney - School of Medicine, BioDiscovery Institute, University of Nottingham, University Park Campus, Nottingham NG7 2RD (UK)

F. Turco, Benjamin Myers - School of Pharmacy, BioDiscovery Institute, University of Nottingham, University Park Campus, Nottingham NG7 2RD (UK)

F. Catrambone - School of Life Sciences, BioDiscovery Institute, University of Nottingham, University Park Campus, Nottingham NG7 2RD (UK)

P. J. Hill - Division of Microbiology, Brewing and Biotechnology, School of Biosciences, University of Nottingham, Sutton Bonington Campus, Nottingham LE12 5RD (UK)

C. Alexander, K. Kovacs - Division of Molecular Therapeutics and Formulation, School of Pharmacy, University of Nottingham, University Park Campus, Nottingham NG7 2RD (UK)

‡Authors contributed equally, joint first author, alphabetical surname order

§Authors contributed equally, joint second author, alphabetic surname order

Materials and Methods

Materials

All chemicals were purchased from reputable suppliers and used without further purification unless stated otherwise. N,N-dimethylacrylamide (DMA), N-acryloylmorpholine (NAM), N-hydroxyethyl acrylate (HEA), diacetone acrylamide (DAA), 2-(2-Carboxyethylsulfanylthiocarbonylsulfanyl)propionic acid) (CTA), glucose, glucose oxidase (GOx), iron(II)chloride hydrate $FeCl_2.4H_2O$, sodium nitrate (NaNO3) and Dulbecco's phosphate buffered saline (DPBS) (without $CaCl_2$ and $MgCl_2$, filtered, suitable for cell culture) were purchased from Sigma Aldrich. Iron(III)chloride hexahydrate (FeCl3.6H2O) ≥98% was purchased from Scientific Laboratory Supplies. VA-044 was purchased from Alpha laboratories. 2-(((butylthio)carbonothioyl)thio)propanoic acid was synthesised as previously reported.[1] For bacteria growth Lysogeny broth (LB) was used.

Methods

Instrumentation

^1H NMR spectra were recorded at room temperature on a 400 MHz (Bruker DPX400 Ultrashield) using deuterated solvents (D_2O). NMR spectra were analysed using MestReNova 11.0.0-17609 2016 Mestrelab Research S.L.

DMF Size Exclusion Chromatography (SEC) was performed on Polymer Laboratories PL50+ system fitted with refractive index (RI) detector, two Agilent PLgel Mixed-D columns and a PLgel guard column eluted with DMF + 0.1% LiBr (w/w). Molecular weight (M_n) and polydispersity ($Đ$) were calculated according to PMMA narrow standards (2,200-0.8 kDa) using Agilent EasyVial calibrants fitted with a cubic function to correlate retention time and molar mass using Cirrus GPC software. Polymer samples were made by dissolving 3 mg/mL pure polymer in 1 mL DMF + 0.1% LiBr. 100 µL samples were injected and eluted at 1 ml/min for 30 min.

Aqueous SEC was performed on an Agilent 1200 system fitted with an RI and UV-vis detector set to 309 nm, Agilent two PL aquagel-OH column and one aquagel guard column eluted with 0.1 M $NaNO_3$ eluent. Molecular weight (M_n) and polydispersity ($Đ$) were calculated according to PEG narrow standards (1,500-0.105 kDa) using Agilent EasyVial calibrants fitted with a cubic function to correlate retention time and molar mass. Polymer samples were made by dissolving 3 mg/mL pure polymer in 0.1 M $NaNO_3$. 50 µL samples were injected and eluted at 1 ml/min for 30 min.

Dynamic light scattering (DLS) measurements were performed using a Malvern Nano Zetasizer (Malvern Instruments, Malvern UK). Data was processed using dispersion technology software (v 7.13, Malvern instruments, Malvern UK) using the multiple narrow modes algorithm based upon a non-negative least square fit to calculate the hydrodynamic radius and the polydispersity index (PD). The Z-average is an intensity-based overall average hydrodynamic size based on a specific fit to the raw correlation function data. The PDI is a measure of the degree of polydispersity of the sample.

TEM analysis was carried out on a JEOL 2100Plus equipped with a Gatan Ultrascan 1000 XP camera. Nanoparticle samples were prepared at a concentration of 1 mg/mL in deionised water and spotted on grids (carbon film on a copper mesh) which were precleaned by a 5 second oxygen/argon plasma clean (Fischione model 1020 Plasma cleaner).

Experimental information

Bacterial culture

C. metallidurans CH34 type strain was purchased from German Collection of Microorganisms and Cell Cultures, Braunschweig, Germany (DSMZ 22839) and routinely grown by directly inoculating 5 mL LB media with the culture stock stored at -80°C. Cultures were grown aerobically, at 30°C in a shaking incubator at 200 rpm overnight (18 hours). Overnight grown cultures were used to inoculate 20 mL LB media to a starting OD_{600nm} = 0.1 and grown at 30°C shaking to an OD_{600nm} ~1. In preparation for the polymerisation, the cultures were centrifuged (6000 rpm, 10 minutes) and the pellets washed twice with

PBS (10 mL) via centrifugation. Each of the washed pellets were re-suspended in 4 mL PBS. 1 mL ($3.4x10^{10}$ CFU ml^{-1}) was added to the polymerisation reaction mixture (1 mL) to give total *C. metallidurans* count of ~$3.4x10^{10}$ in the 2 mL volume, or $1.7x10^{10}$ CFU mL^{-1} (per mL). When comparing live and dead cultures, live cultures were stored on ice until used in polymerisation. Dead bacterial cultures were heat killed by incubating at 70°C for 20 minutes.

Toxicity of water-soluble monomers to C. metallidurans

Minimum inhibitory concentration *(*MIC) experiments of the monomers used in this work were carried out using microplate reader with 96-Well standard microplates (Costar 3363), measuring the OD600 nm every 30 minutes for up to 24 hours. An overnight bacterial culture was diluted to an OD600 0.01 and incubated at 37°C, 200 rpm for an additional 2 h. The culture was then diluted back to an OD600 of 0.005. Monomers were prepared at a range of concentrations through serial dilution. In 96-well plates 50 µl of cell culture was added to 50 µl of test compounds. Initial OD600 readings were taken using a 96-well plate reader (Infinite M Nano, TECAN), and plates were then left to incubate overnight; 24 h at 37°C. OD600 readings were taken every 30 min and MIC values were determined as the lowest concentration of test compound to inhibit growth; whereby OD600 value did not increase.

Bacterially initiated polymerisation procedure

Polymerisations were conducted with the following general procedure targeting DP400. DMA (100 mM), CTA (0.24 mM), Glu (100 mM), GOx (0.25 µM), Fe^{3+} (7 µM) and 1 mL of bacteria were mixed together to a total volume of 4 mL and shaken overnight at 30 °C. To quench the polymerisation, the polymerisation mixture was centrifuged at 4000 g to pellet *C.metallidurans* cells and the supernatant was lyophilised. Conversions were assessed through ^1H NMR spectroscopy by dissolving the remaining powder in deuterium oxide for analysis. Variations to this procedure are noted below in Table S3. For kinetics experiments, polymerisations were scaled up to a total volume of 16 mL and shaken at 30 °C. At varying timepoints, 4 mL aliquots were removed and treated as above. Polymerisations were also carried out in the absence of some components i) with bacteria but no $FeCl_3.6H_2O$; ii) without any bacteria; and iii) with no bacteria and no $FeCl_3.6H_2O$. Variations to this procedure are noted below in Table S3.

Synthesis of $pDMA_{75}$

$pDMA_{75}$ was synthesised as follows. 2-(((butylthio)carbonothioyl)thio)propanoic acid (134.7 µmol), DMA (10.1 mmol) and VA-044 (10.7 µM) were added to total volume of 3.4 mL water and stirred at 70 °C for 2 hours. Monomer conversion was found to be >99% determined by ^1H NMR spectroscopy in d_6-DMSO. The pure polymer was isolated by lyophilization.

Polymerisation-induced self-assembly

Diacetone acrylamide (9.9 mM), $pDMA_{75}$ (22.7 µM), glucose (100 mM), glucose oxidase (0.25 µM), $FeCl_3.6H_2O$ (7 µM) and 1 mL of *C. met* culture (1.7 $x10^{10}$ CFU) were mixed to a total volume of 4 mL and shaken overnight at 30 °C. Samples were centrifuged down to remove bacteria and the supernatant was kept for DLS and TEM analysis.

Recycling C. met culture in successive polymerisations

Polymerisations were set up as described above and shaken overnight at 30 °C. Following 24 hours of incubation, the suspensions were separated by centrifugation as above and the supernatant was collected for further analysis. The bacterial pellet was resuspended in 1 mL DPBS and another mixture of DMA, CTA, $FeCl_3.6H_2O$, GOx, and Glucose as above were added to the tube. This procedure was repeated so that three successive polymerisations were performed.

Supplementary information

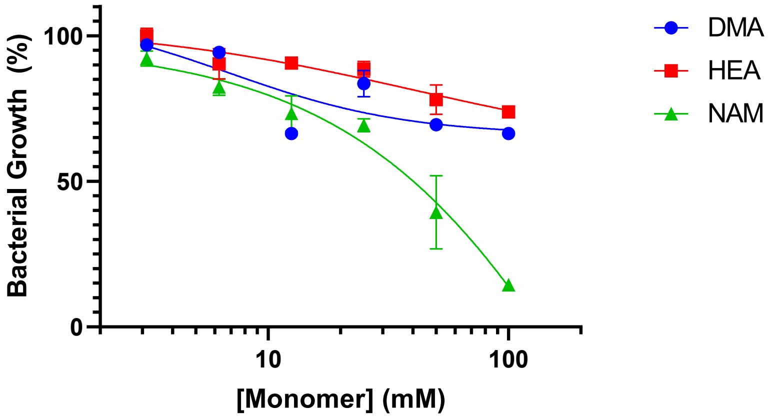

Figure S1. Toxicity study to observe the effect of monomers DMA, HEA and NAM on the growth of *C.metallidurans* at different concentrations following 24 hours of incubation. Growth was calculated using OD_{600} values as compared to an untreated bacteria sample

Table S1. MICs of monomers, DMA, HEA and NAM, towards *C. met* metabolism

	MIC_{50} (mM)		
	DMA	HEA	NAM
C. met	> 100	> 100	40

Table S2. Colony forming units (CFU) in the 1 mL culture added to each polymerisation

Culture	Heat Treated	CFU (mL^{-1})
Live	No	$1.7x10^{10}$
Dead	Yes	368

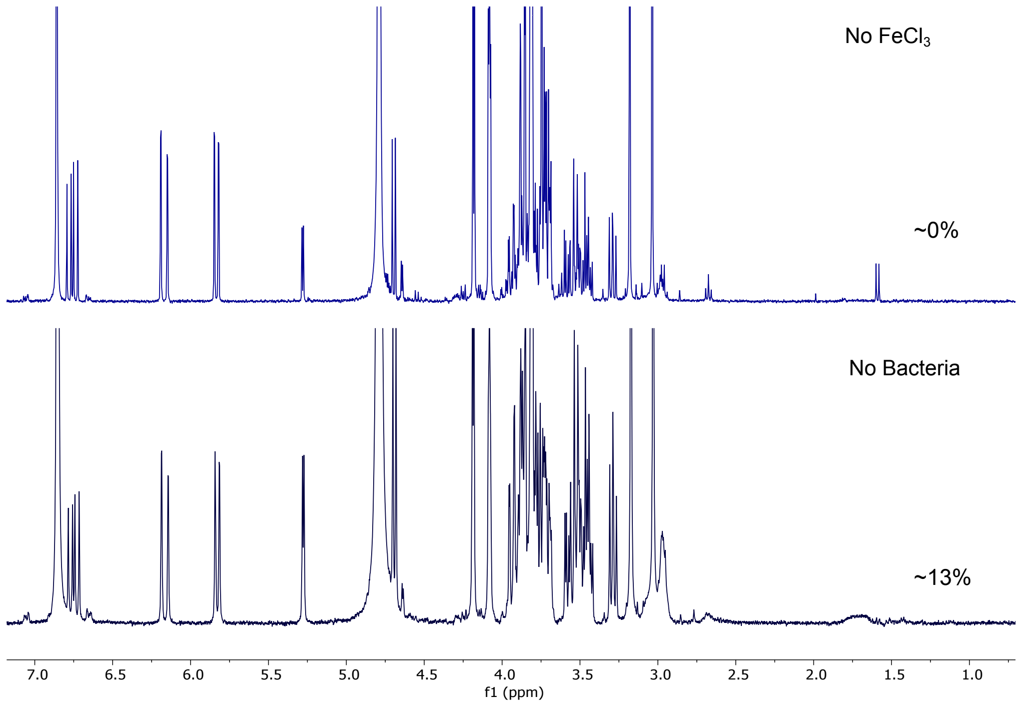

Figure S2. ^{1}H NMR (400 MHz, D_2O) Time 24 hours showing no/little polymer formation. Top; Bacterial-Fenton-GOx-RAFT (b-FG-RAFT) without any Iron source. Bottom; (b-FG-RAFT) without any bacteria.

Table S3. Results recorded for polymerisations without addition of CTA, following variation of concentration of Fe^{3+}, reaction time and monomer. Standard conditions were followed to target DP400 (100 mM monomer for DMA and HEA, 25 mM monomer for NAM, 0.24 mM CTA, 100 mM Glu, 0.25 µM GOx, 7 µM Fe^{3+} and 1 mL of bacteria) unless stated otherwise.

Monomer	Reaction Conditions	Conv. (%)[a]	$M_{n,th}$ (g mol^{-1})[b]	$M_{n,\ SEC}$ (g mol^{-1})[c]	Đ[c]
DMA	No CTA	95.9	-	451,300(i)	2.12
DMA	7 µM Fe	44.3	17,800	20,700(i)	1.28
DMA	70 µM Fe	58.9	23,600	25,100(i)	1.49
DMA	700 µM Fe	66.2	26,500	31,500(i)	1.46
DMA	7000 µM Fe	8.7	3,700	126,500(i)	2.11
DMA	1 h	28.0	8,600	20,500(ii)	1.40
DMA	2 h	36.1	11,500	20,300(ii)	1.42
DMA	24 h	41.0	13,300	22,700(ii)	1.11
HEA	-	36.6	17,100	31,800(ii)	1.56
NAM	-	40.3	23,000	246,500(ii)	1.58
(NAM + DMA)	12.5 mM NAM + 50 mM DMA	62.6	13,500	12,100(ii)	1.21
Macro(DMA)	*Synthesised through conventional RAFT*	>99%	7,800	8,000(ii)	1.15
Macro(DMA) + DMA	2.7 µM Macro(DMA) + 100 mM DMA	49.1	27,700	20,200(ii)	1.87
DMA	DP100	58.6	6,100	6,300(ii)	1.17
DMA	DP400	32.2	13,000	20,700(ii)	1.3
DMA	DP800	44.3	35,400	55,800(ii)	1.7

[a] Estimated using of ^1H NMR from comparison of monomer (CH acrylate): polymer (CH_2 backbone) integrals. [b] M_n^{th} = ($Mr_{monomer}$)* 100* conversion) + 254 Da. [c]Calculated using SEC ((i)DMF with RI detector; (ii) aqueous with UV detector).

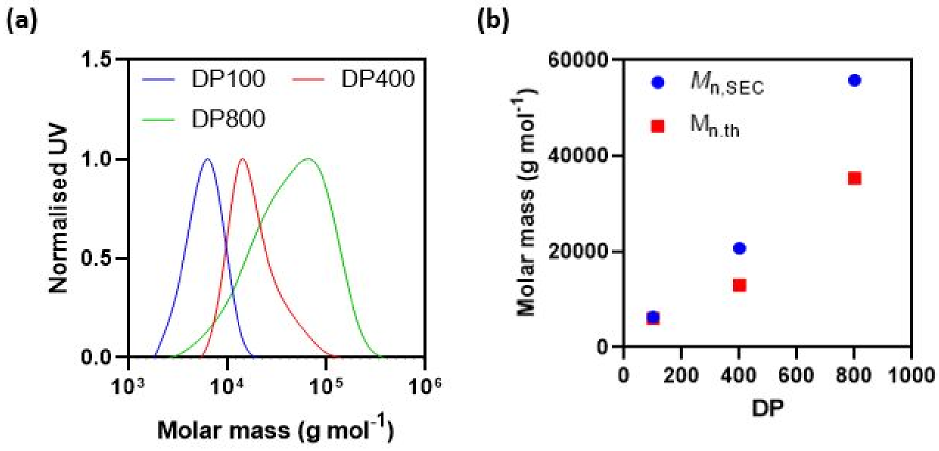

Figure S3. (a) SEC (aqueous, UV detector) of pDMA prepared at varying target DP. **(b)** Comparison of $M_{n,SEC}$ (aqueous, UV signal) and $M_{n,th}$ as a function of target DP.

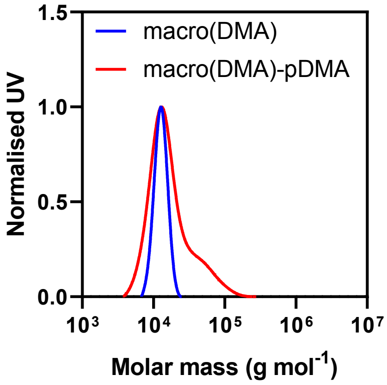

Figure S4. SEC (aqueous, UV detector) of a chain extension carried out using a previously prepared macro(DMA).

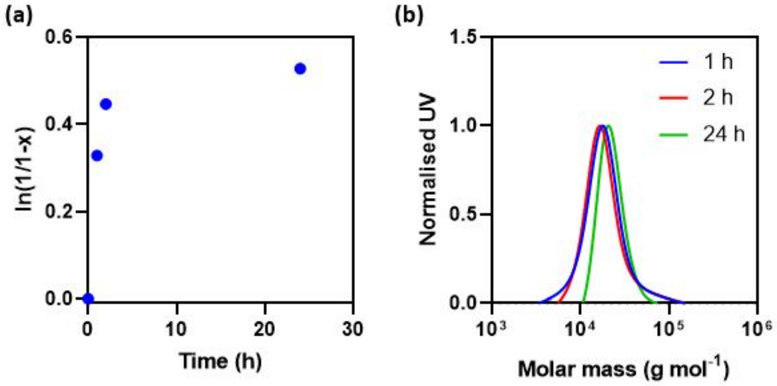

Figure S5. (a) Kinetics plot of ln(1/1-x) against time of polymersiation, where x is the conversion as determined by ^{1}H NMR (400 MHz, D_2O) **(b)** SEC (aqueous, UV signal) overlay of polymers produced following variation of reaction time

References

1. Gurnani, P.; Lunn, A. M.; Perrier, S., Synthesis of mannosylated and PEGylated nanoparticles via RAFT emulsion polymerisation, and investigation of particle-lectin aggregation using turbidimetric and DLS techniques. *Polymer* **2016,** *106*, 229-237.
